# The Age Structure, Stringency Policy, Income, and Spread of Coronavirus Disease 2019: Evidence From 209 Countries

**DOI:** 10.3389/fpsyg.2020.632192

**Published:** 2021-02-12

**Authors:** Faik Bilgili, Munis Dundar, Sevda Kuşkaya, Daniel Balsalobre Lorente, Fatma Ünlü, Pelin Gençoğlu, Erhan Muğaloğlu

**Affiliations:** ^1^Faculty of Economics and Administrative Sciences, Department of Economics, Erciyes University, Melikgazi, Turkey; ^2^Faculty of Medicine, Internal Medical Sciences, Department of Medical Genetics, Erciyes University, Talas, Turkey; ^3^Department of Law, Justice Vocational College, Erciyes University, Talas, Turkey; ^4^Faculty of Social Sciences, Department of Public Finance, University of Castilla La Mancha, Cuenca, Spain; ^5^Faculty of Economics and Administrative Sciences, Department of Economics, Erciyes University, Talas, Turkey; ^6^Erciyes University Research and Application Center of Kayseri, Melikgazi, Turkey; ^7^Faculty of Managerial Sciences, Department of Economics, Abdullah Gül University, Kayseri, Turkey

**Keywords:** COVID-19 cases, income, stringency policy, COVID-19 deaths, 70-aged people, median aged people, 65-aged people, government responses

## Abstract

This article aims at answering the following questions: (1) What is the influence of age structure on the spread of coronavirus disease 2019 (COVID-19)? (2) What can be the impact of stringency policy (policy responses to the coronavirus pandemic) on the spread of COVID-19? (3) What might be the quantitative effect of development levelincome and number of hospital beds on the number of deaths due to the COVID-19 epidemic? By employing the methodologies of generalized linear model, generalized moments method, and quantile regression models, this article reveals that the shares of median age, age 65, and age 70 and older population have significant positive impacts on the spread of COVID-19 and that the share of age 70 and older people in the population has a relatively greater influence on the spread of the pandemic. The second output of this research is the significant impact of stringency policy on diminishing COVID-19 total cases. The third finding of this paper reveals that the number of hospital beds appears to be vital in reducing the total number of COVID-19 deaths, while GDP per capita does not affect much the level of deaths of the COVID-19 pandemic. Finally, this article suggests some governmental health policies to control and decrease the spread of COVID-19.

## Introduction

Throughout human history, societies have witnessed numerous epidemic diseases (such as the Black Death, Spanish influenza, SARS, MERS, Ebola, and Zika) that have led to political, social, economic, and technological changes (Garrett, 2007; UN, 2014; Baldwin and Mauro, 2020; Ojo, 2020). The last experienced pandemic is coronavirus disease 2019 (COVID-19), which continues to have a profound global impact on the world. COVID-19, which has been declared a pandemic by the World Health Organization (WHO) on March 11, 2020, is a virus epidemic that has affected the whole world severely since its occurrence in Wuhan, China, in December 2019 (Sohrabi et al., 2020).

As expressed by many eminent scientists and especially by the WHO, the contagiousness of the virus causing COVID-19 disease was high. Over seven million COVID-19 cases and more than 400,000 deaths were reported to WHO by June 2020 (WHO, 2020a). The distribution of total COVID-19 cases around the world was as follows: America (0.478), Europe (0.327), Eastern Mediterranean (0.094), South-East Asia (0.054), Western Pacific (0.027), and Africa (0.020) (WHO, 2020a). In addition, the proportion of deaths was 0.457 (Europe), 0.455 (America), 0.037 (Eastern Mediterranean), 0.026 (South-East Asia), 0.018 (Western Pacific), and 0.008 (Africa) (WHO, 2020a). This virus is novel and no treatment methods such as vaccines or drugs still exist. The virus has inevitably caused severe uncertainty and anxiety in the whole world.

During the epidemic, various economic, and non-economic measures have been taken by governments all over the world (Bruin et al., 2020; Delivorias and Scholz, 2020; Nicola et al., 2020). By following these precautions, the economic and social costs of the pandemic are still being tried to be minimized by eliminating the negative effects of the epidemic. The costs of these pandemic outbreaks in countries can be classified into two groups: (i) direct costs due to emergent need for intervention to outbreaks like medical testing, diagnosis, treatment, and equipment costs; or (ii) indirect costs related to economical production and productivity losses due to the epidemic (Demir, 2020). These costs are expected to have several effects on social and economic life in both the short- and long-term (Ali and Alharbi, 2020; Nicola et al., 2020). These social influences resulted in poverty, income inequality, less access to essential public services, distorted social psychology, and limited social communication. At the same time, the economic effects caused undesirable changes in macroeconomic indicators. According to the estimates of ILO (2020), it is expected that the increase in global unemployment will be 5.3 million for the best scenario and 24.7 million for the worst scenario.

Considering the main effects of the COVID-19 pandemic, demographic characteristics (like age, gender, and others) come to the fore in the distribution of these effects in societies. In studies examining the effects of the COVID-19 pandemic, they have been focused on various topics such as mortality rate (Bonanad et al., 2020; Bulut and Kato, 2020; De Paz et al., 2020; Jin et al., 2020; Liu et al., 2020a; Meo et al., 2020; Roberton et al., 2020), psychological effects (Ahmed et al., 2020; Cao et al., 2020; Gorrochategi et al., 2020; Shrira et al., 2020), and the spread of the disease (Banerjee et al., 2020; Chen Z. et al., 2020; DeBiasi et al., 2020; Dolores De Luca et al., 2020; Huang et al., 2020; Li et al., 2020; Liu et al., 2020b,c; Meo et al., 2020; Mustafa and Selim, 2020; Palmieri et al., 2020). Two main findings have been reached: (a) mortality rates associated with COVID-19 are higher in individuals over 65 years of age than in younger individuals, and (b) by age groups, the impacts of the intensity of COVID-19 disease on community psychology vary. As age increases, negative psychological effects such as stress, anxiety, and depression have occurred more among individuals over 65 years old. Briefly, the specific features of the virus such as high contagion rate and risk of death have different effects primarily on populations with different age structures. For instance, the contagion rate of COVID-19 is higher in children and young age groups, while the mortality rates are higher in older people, especially those over 65 years.

Although there exist several studies examining the effects of COVID-19 from different perspectives in the relevant literature, econometrical analyses estimating the relationship between age structure and the pandemic are limited. Additionally, one may claim that majority of the existing literature follows some comparison analyses and/or some econometric analyses in which heterogeneous structure and quantile structure of the data are not considered.

In this regard, in this research, the outputs from 209 countriesare expected to reveal unbiased, efficient, and consistent results to explore the basic determinants of COVID-19 cases and deaths. The purpose of this article is to determine the impact of age structure and government policy in response to the COVID-19 outbreak (stringency policy) in 209 countries in the world by applying the generalized linear model (GLM), generalized moments method (GMM), and quantile regression model (quantile).

The article is organized as follows: after the section “Introduction,” the second section contains the “Data and Methodology.” The third section introduces the “Study Findings.” The fourth and fifth sections will reveal the “Discussion and Conclusion,” respectively.

## Data and Methodology

### Data

The data, data source, and the list of cross-sections (countries) are given in Appendix Tables A1, A2. The variables are total cases per million; total deaths per million; the shares of the median age population, age 65 population, and age 70 and older population; stringency index; GDP per capita; and the number of hospital beds, respectively. The stringency index (or stringency policy index) indicates the governments’ policies in response to the COVID-19 outbreak and contains information on containment and closure policies, such as school closures and restrictions in movement; economic policies, such as income support to citizens or provision of foreign aid; and health system policies such as the COVID-19 testing regime or emergency investments into healthcare.

### Methodology

This article employs the methodologies of GLM, GMM, and quantile regression to yield the relevant model estimations by following the functions given in Eqs 1–6.

(1) Total⁢COVID-19⁢cases=α⁢10+α⁢11⁢(Median⁢age⁢population)+α⁢12⁢(Stringency⁢policy)+e

(2) Total⁢COVID-19⁢cases=α⁢20+α⁢21⁢(Age⁢ 65⁢and⁢older⁢population)+α⁢23⁢(Stringency⁢policy)+ϕ

(3) Total⁢COVID-19⁢cases=α⁢30+α⁢31⁢(Age⁢ 70⁢and⁢older⁢population)+α⁢32⁢(Stringency⁢policy)+η

(4) Total⁢COVID-19⁢deaths=α⁢40+α⁢41⁢(Median⁢age⁢population)+α⁢42⁢(Total⁢COVID-19⁢cases)+α⁢43⁢(GDP⁢per⁢capita)+α⁢44⁢(#⁢Hospital⁢beds)+s

(5) Total⁢COVID-19⁢deaths=α⁢50+α⁢51⁢(Age⁢ 65⁢and⁢older⁢population)+α⁢52⁢(Total⁢COVID-19⁢cases)+α⁢53⁢(GDP⁢per⁢capita)+α⁢54⁢(#⁢Hospital⁢beds)+ŗ

(6) Total⁢COVID-19⁢deaths=α⁢60+α⁢61⁢(Age⁢ 70⁢and⁢older⁢population)+α⁢62⁢(Total⁢COVID-19⁢cases)+α⁢63⁢(GDP⁢per⁢capita)+α⁢64⁢(#⁢Hospital⁢beds)+ς

Where *e*, ϕ, η, *s*, *ŗ*, and *ς* denote the residuals of the estimated models. The age variables represent the shares of median age, age 65, and age 70 and older people in the population. #Hospital beds indicate the number of hospital beds.

Generalized moments method, which can be considered as a nested estimation method (maximum likelihood, least squares, instrumental variables, and two-stage least squares), is one of the most preferred methods of econometric analysis in recent years. The GMM allows us to estimate the moments of a population distribution relative to the moments predicted from a particular sample (Burguete et al., 1982; Hansen, 1982; Asteriou and Hall, 2015).

The Arellano and Bond (1991)-type one-step estimator uses the identity matrix as a weighting matrix (Cameron and Trivedi, 2005). The one-step GMM uses the weighting matrix and the estimator as defined in Eqs 7 and 8. It can be shown that the estimator is the optimal panel GMM estimator as *u*_*i*_|*Z*_*i*_. is iid [0,σ^2^*I*_*t*_].

(7) $$W_{N}={\left[\sum_{i}Z_{i}^{{}^{\prime }}\,Z_{i}\right]}^{-1}={\left[Z^{{}^{\prime }}\,Z\right]}^{-1}$$

(8) $${\hat{\beta }}_{2\,S\,L\,S}={\left[X^{\prime }\,Z\,{\left(W\right)}^{-1}\,Z^{\prime }\,X\right]}^{-1}\,X^{\prime }\,Z\,{\left(W\right)}^{-1}\,Z^{\prime }\,y$$

Normal distribution plays an important role in linear and nonlinear regression models. GLMs are those of the nonlinear models without normal distribution assumption in the dependent variable (Collins, 2008).

Generalized linear model consists of three components: distribution of the dependent variable, systematic part with linear estimators, and link function (De Jong and Heller, 2008). The probability density function of the response variable from the exponential distribution family can be written as shown in Eq. 9.

(9) $$f\,\left(x;\varphi ,\omega \right)=m\,\left(x,\omega \right)\,\text{exp}\,\left\{\frac{x\,\varphi -n\,\left(\varphi \right)}{k\,\left(\omega \right)}\right\}$$

Where *k*(.),*n*(.), and *m*(.) are certain functions. The term φ is the natural location parameter. The term ω is the scale parameter. In Eq. 9, the term φ is the canonical parameter, the term ω is the propagation parameter, and *m*(*x*,ω) is a known function. The choice of the functions *n*(φ) and *m*(*x*,ω) determines the actual probability function such as the binomial, normal, or gamma.

Notably, in the case of heterogeneity structure in the variance, the OLS analyses might fail to estimate the coefficients efficiently and consistently. Under heterogeneity structure in the variance, we need alternative regression models such as quantile regression models in which heterogeneity structure and quantile structure of the data are considered (John and Nduka, 2009; Katchova, 2013). Quantile regression aims to estimate the conditional median or other quantiles, such as the 10th quantile, 25th quantile, 75th quantile, and 90th quantile of the response variable (Ong et al., 2015).

Quantile regression determines the model for the selected percentiles in the conditional distribution of the dependent variable (Palma et al., 2020). Hence, traditionally, the linear regression model is expressed as follows:

(10) $$z_{i}=\alpha_{0}+\alpha_{1}\,y_{i\,1}+\cdots +\alpha_{p}\,y_{i\,p}\;i=1,\ldots ,n$$

Parameter *p* in Eq. 10 shows the number of parameters in the equation. The term*i* is equal to the number of data points. As a similar formation to the linear regression model, the quantile regression model equation for the τ-th quantile can be written as follows:

(11) $$Q_{\tau }\,\left(z_{i}\right)=\alpha_{0}\,\left(\tau \right)+\alpha_{1}\,\left(\tau \right)\,y_{i\,1}+\cdots +\alpha_{p}\,\left(\tau \right)\,y_{i\,p}\;i=1,\ldots ,n$$

Thus, alpha coefficients have become functions that change depending on the quantile (Dye, 2020).

## Study Findings

**FIGURE 1:**
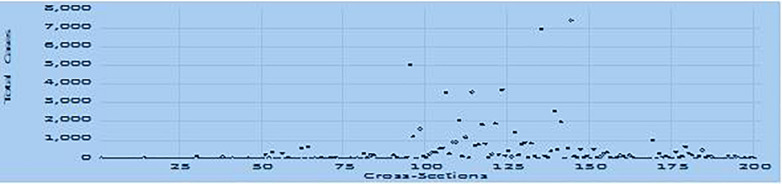
Total COVID-19 cases per million (April 2, 2020).

**FIGURE 2:**
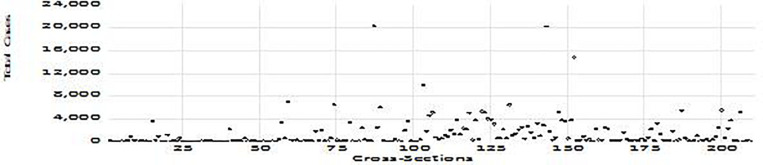
Total COVID-19 cases per million (June 2, 2020).

**FIGURE 3:**
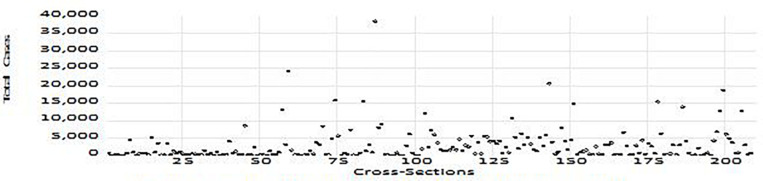
Total COVID-19 cases per million (August 2, 2020).

The cases and deaths denote the cases and deaths from the COVID-19 pandemic. The cross-sections have been chosen based on the availability of data for 209 countries as of (a) April 2, 2020; (b) June 2, 2020; and (c) August 2, 2020, and depicted in Figures 1–3, respectively.

Table 1 shows the impacts of different age structures (shares of median age, age 65, and age 70 and older people in the population) on COVID-19 total cases per million as of April 2, 2020, in 209 countries. The second and third columns exhibit the GLM and GMM estimations. Besides GLM and GMM, due to the heteroscedasticity structure of the data, we have decided to follow quantile regression analyses to observe the parameter estimations at different quantiles of the data as the routine recommended in Katchova (2013).

**TABLE 1:** The impact of age structure on COVID-19 total cases in April 2020.

**Independent variables**	**GLM^1,2^**	**GMM^3,4,5^**	**Quantile (25)^6,7,8^**
*Median age*	7.91 (1.13) [6.98] {0.00}	6.80 (1.40) [4.84] {0.00}	0.35 (0.26) [1.34] {0.18}
			**Quantile (50)^6,7,8^**
			1.60 (0.28) [5.66] {0.00}
			**Quantile (75)^6,7,8^**
			5.88 (0.98) [5.98] {0.00}
*Aged-65_older*	27.40 (3.19) [8.58] {0.00}	2 3.46 (3.70) [6.32] {0.00}	2.41 (1.14) [2.11] {0.03}
			**Quantile (50)^6,7,8^**
			8.97 (1.39) [6.40] {0.00}
			**Quantile (75)^6,7,8^**
			24.51 (4.96) [4.93] {0.00}
*Aged-70_older*	43.17 (4.85) [8.89] {0.00}	37.47 (5.71) [6.56] {0.00}	3.73 (1.79) [2.08] {0.03}
			**Quantile (50)^6,7,8^**
			15.42 (2.19) [7.01] {0.00}
			**Quantile (75)^6,7,8^**
			42.66 (5.66) [7.53] {0.00}

*^1^By Newton-Raphson-Marquardt steps. ^2^The coefficient covariance is computed using observed Hessian. ^3^Estimation weighting matrix: HAC (Bartlett kernel, Newey-West fixed bandwidth = 5.0000). ^4^Standard errors and covariance are computed following the estimation weighting matrix. ^5^Instrument specification: total-cases-per-million (−1). ^6^Sparsity methods: Kernel (Epanechnikov) using residuals. ^7^Bandwidth methods: Hall-Sheather. ^8^Estimation successfully identifies the unique optimal solution. () shows standard errors, and {} denotes probability values.*

In Table 1, GMM, GLM, and quantile (at 50 and 75 quantiles) regression analyses exhibit significant positive contributions of shares of median age, age 65, and age 70 and older people in the population to the total COVID-19 cases. The quantile estimations did not find a significant impact of the median age population on total cases at the 25th quantile. The quantile 50th (median) estimation reveals that the unit increase in the share of median age people caused the total number of COVID-19 cases to increase by 1.61 units. Moreover, the COVID-19 cases will be 5.88 units (cases) more due to a unit increase in the share of median age people at the 75th quantile. Table 1 reveals that upon a 1-unit increase in the share of median age in the population, the number of total COVID-19 per million increased significantly by 7.91 units (by GLM estimation) and by 6.80 units (by GMM estimation).

Table 1 reaches the evidence that an increase in the percentage of people aged 65 and older increased the total number of COVID-19 cases by 2.42 units at the 25th quantile. The quantile 50th (median) estimation exhibits that a1-unit increase in the percentage of peopleaged 65 and older increased the total number of COVID-19 cases by 8.97 units. The COVID-19 cases will be 24.51 units (cases) more due to a unit increase in the percentage of people aged65 and older at the 75th quantile.

Table 1 indicates the impacts of people aged 70 and over on the total COVID-19 cases. All three estimation models state that the higher the share of people aged70 and over in the population, the more the COVID-19 cases will be. The quantile regression results give more details on the influence of age structure on the number of cases. The quantile estimation results in Table 1 show that if any country has an additional one more unit of the share of the population aged 70 and over, that country will have 3.73 more units of the total cases (at the 25th quantile), 15.42 more units of the total cases (at the 50th quantile), and 42.66 more cases (at the 75th quantile of the total case data) as of April 2, 2020, in 200 countries.

Table 2 yields the influences of the share of the median age population on the COVID-19 pandemic as of June 2, 2020, in 209 countries. GLM, GMM, and quantile estimations reached significant positive coefficients of the share of median age on COVID-19 cases. GLM and GMM estimations are 39.13 and 37.81, respectively. The predictions of quantiles (25), (50), and (75) are 3.94, 12.07, and 46.62, respectively.

**TABLE 2:** The impact of age structure on COVID-19 total cases on June 2020.

**Independent variables**	**GLM^1,2^**	**GMM^3,4,5^**	**Quantile (25)^6,7,8^**
*Median age*	39.12 (4.65) [8.41] {0.00}	37.81 (5.10) [7.40] {0.00}	3.93 (1.68) [2.33] {0.02}
			**Quantile (50)^6,7,8^**
			12.07 (1.92) [6.28] {0.00}
			**Quantile (75)^6,7,8^**
			46.62 (9.38) [4.96] {0.00}
*Aged-65_older*	106.08 (14.4) [7.33] {0.00}	127.1 (18.3) [6.93] {0.00}	18.49 (5.74) [3.21] {0.00}
			**Quantile (50)^6,7,8^**
			45.65 (7.55) [6.04] {0.00}
			**Quantile (75)^6,7,8^**
			143.04 (23.68) [6.04] {0.00}
*Aged-70_older*	161.1 (22.19) [7.26] {0.00}	157.7 (22.72) [6.93] {0.00}	30.41 (7.99) [3.80] {0.00}
			**Quantile (50)^6,7,8^**
			71.79 (13.13) [5.46] {0.00}
			**Quantile (75)^6,7,8^**
			214.8 (32.12) [6.68] {0.00}

*^1^By Newton-Raphson-Marquardt steps. ^2^The coefficient covariance is computed using observed Hessian. ^3^Estimation weighting matrix: HAC (Bartlett kernel, Newey-West fixed bandwidth = 5.0000). ^4^Standard errors and covariance are computed following the estimation weighting matrix. ^5^Instrument specification: total-cases-per-million (−1). ^6^Sparsity methods: Kernel (Epanechnikov) using residuals. ^7^Bandwidth methods: Hall-Sheather. ^8^Estimation successfully identifies the unique optimal solution. () shows standard errors, and {} denotes probability values.*

The quantile regressions show that the impacts of the share of people aged 65 (share of peopleaged 70) on the expansion of COVID-19 are 18.49 (30.41) at the 25th quantile and 143.04 (214.88) at the 75th quantile on June 2, 2020 (Table 2).

Table 3 yields the magnitudes of the responses of total COVID-19 cases per million to the unit increase in shares of median age, age 65, and age 70 in the population as of August 2, 2020, in 208 countries. In Tables 1–3, the estimation results have similar remarks depicting that the impact of age structure on dispersing of the COVID-19 epidemic (i) tends to increase significantly from median age to age 65 and from age 65 to age 70+ in the population; (ii) tends to increase from April 2, 2020, to August 2, 2020; and (iii) tends to increase considerably from lower quantile to higher quantile of the data for 209 cross-sections.

**TABLE 3:** The impact of age structure on COVID-19 total cases on August 2020.

**Independent var**iables	**GLM^1,2^**	**GMM^3,4,5^**	**Quantile (25)^6,7,8^**
*Median age*	91.91 (10.58) [8.68] {0.00}	84.47 (12.21) [6.91] {0.00}	10.33 (4.94) [2.09] {0.03}
			**Quantile (50)^6,7,8^**
			36.01 (5.78) [6.22] {0.00}
			**Quantile (75)^6,7,8^**
			112.7 (15.49) [7.27] {0.00}
*Aged-65_older*	222.6 (34.15) [6.51] {0.00}	301.7 (49.39) [6.10] {0.00}	40.46 (15.30) [2.64] {0.00}
			**Quantile (50)^6,7,8^**
			120.5 (19.32) [6.23] {0.00}
			**Quantile (75)^6,7,8^**
			335.31 (53.97) [6.21] {0.00}
*Aged-70_older*	333.38 (52.65) [6.33] {0.00}	465.6 (78.30) [5.94] {0.00}	48.05 (24.5) [1.96] {0.05}
			**Quantile (50)^6,7,8^**
			171.4 (28.9) [5.92] {0.00}
			**Quantile (75)^6,7,8^**
			541.9 (83.3) [6.50] {0.00}

*^1^By Newton-Raphson-Marquardt steps. ^2^The coefficient covariance is computed using observed Hessian. ^3^Estimation weighting matrix: HAC (Bartlett kernel, Newey-West fixed bandwidth = 5.0000). ^4^Standard errors and covariance are computed following the estimation weighting matrix. ^5^Instrument specification: total-cases-per-million (–1). ^6^Sparsity methods: Kernel (Epanechnikov) using residuals. ^7^Bandwidth methods: Hall-Sheather. ^8^Estimation successfully identifies the unique optimal solution. () shows standard errors, and {} denotes probability values.*

The impacts of different age structures on COVID-19 total cases in the continents of Europe, America, Asia, and Africa are given in Appendix Table A3. Two main outcomes appear. Firstly, the adverse effects of age structures on total cases in all continents are subject to a prominent increase from median age to age 70 and older people. This output confirms the earlier results given in tables from 1 to 9. Secondly, the negative effects of median age, age 65, and age 70 populations in Europe on COVID-19 cases are greater than the negative effects of the same age groups on COVID-19 cases in the other three continents. The negative effects of these age groups on total cases in the American continent are higher than the negative effects of the same age groups in the other two continents (Asia and Africa). The impact of the COVID-19 pandemic on people with chronic diseases such as hypertension and cardiac insufficiency (Chen Y. et al., 2020; Ip et al., 2020; Polverino et al., 2020; Roncon et al., 2020), diabetes (Bornstein et al., 2020; Guo et al., 2020; Liu et al., 2020c; Zhang et al., 2020c,d), and cancer (Fratino et al., 2020; Zhang et al., 2020a) is more serious. Chronic diseases are especially higher in older adults (Atella et al., 2018; Zhao et al., 2018; NCOA National Council on Aging [NCOA], 2020) because changes in gene expression during aging are seen as one of the most important biological markers of aging and chronic diseases (Mueller et al., 2020).

**FIGURE 4:**
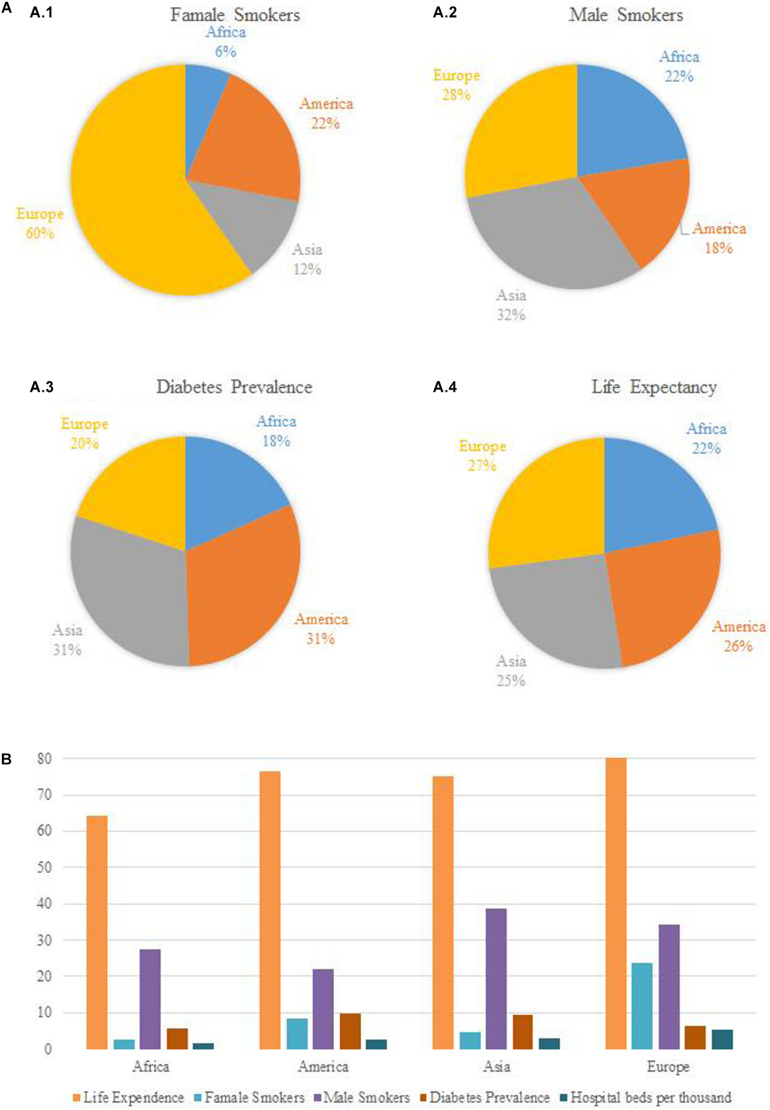
**(A)** Selected indicators (average values) for continents (June, 2020). **(B)** Bar charts.

The average lifetime and population rates in terms of diabetes and smoking might also have a considerable role in the level of the COVID-19 pandemic. In Figures 4A, A.1, A.2 show that the percentages of female smokers in the female populations are relatively higher in Europe and America than those in Asia and Africa and that the percentages of male smokers in the male populations are greater in Asia and Europe than those in Africa and America. The statistical output of high rate of smoking in Europe might be the cause of the higher rate of COID-19 cases in Europe. According to theWHO (2020b), there is evidence that a relationship exists between smoking and increased severity of disease and death in hospitalized COVID-19 patients.

Figure 4A.3 evaluating the prevalence of diabetes, which is one of the chronic diseases, exhibits that the continents with the higher rates in all age groups are America and Asia, respectively. Figure 4A.4 shows that the average life expectancy is relatively higher in the continents of Europe and America than that in Asia and Africa. The longer average lifetime might increase the possibility of having chronic diseases in population density. This interpretation of course needs to be confirmed also by econometric models which can depict the impacts of a lifetime on COVID-19 cases in each continent separately.

In addition to the pie charts (Figure 4A; from Figures 4A.1–A.4), the bar chart (Figure 4B) has been also added to the paper in order for the potential reader to be able to follow the manuscript clearly and fluently.

As a possible determinant of COVID-19 total cases, we also considered the stringency index (government measures/policies in response to the COVID-19 outbreak) for each cross-section in the panel data. The stringency index (stringy policy) exhibits the composite measure based on COVID-19 response indicators including school closures, workplace closures, and travel bans as explained in Appendix Table A1. Table 4 reveals the role of the countries’ stringency policies in determining the spread of total COVID-19 cases in panel data as of April 2020 and June 2020, respectively.

**TABLE 4:** he impact of age structure and stringency policy on spread of COVID-19 cases.

**Independent Variables**	**Quantile (25)**	**Probability**	**Quantile (50)**	**Probability**	**Quantile (75)**	**Probability**
(In terms of April 2, 2020)*						
***Median age***	1.19	0.11	5.70	0.000	20.32	0.0002
*Stringency index*	−0.27	0.21	−1.34	0.003	−4.47	0.0011
(In terms of June2, 2020)**						
***Median age***	8.03	0.08	24.29	0.0001	90.01	0.0000
*Stringency index*	−1.54	0.36	−4.82	0.0244	−18.31	0.0007
(In terms of April 2, 2020)*						
***Aged-65_older***	6.29	0.0066	13.81	0.0000	38.87	0.0002
*Stringency index*	−0.29	0.0538	−0.53	0.0000	−1.28	0.0081
(In terms of June 2, 2020)**						
***Aged-65_older***	23.92	0.0154	61.30	0.0007	160.63	0.0000
*Stringency index*	−0.61	0.5313	−1.40	0.2607	−3.46	0.0391
(In terms April 2, 2020)*						
***Aged-65_older***	8.44	0.0101	21.57	0.0000	60.56	0.0000
*Stringency index*	−0.22	0.0924	−0.48	0.0004	−1.15	0.0001
(In terms of June 2, 2020)**						
***Aged-70_older***	36.70	0.0076	97.57	0.0017	246.78	0.0000
*Stringency index*	−0.53	0.5286	−1.23	0.3221	−2.85	0.0304

Because of the absence of the stringency index data, August 2020 outputs are not available. (*) As of April 2, 2020, in 159 countries, (**) as of June 2, 2020, in 160 countries.

Since the stringency index is not available during August 2020, we could not observe its impact on total cases during August. The GLM and GMM outputs yielded a negative sign but insignificant effects of stringency policy on total cases (to save space, we do not place these outputs here). Quantile regressions, on the other hand, revealed significant negative estimated impacts of stringency policy on COVID-19 total cases. The tables underline that (a) the increase in median age, age 65, and age 70 and older population will increase the COVID-19 cases during both April and June 2020; (b) the stringency policy is found significant to lower COVID-19 cases at the 25th, 50th, and 75th quantiles during April 2020; and (c) the stringency policy is important to diminish COVID-19 cases at the 75th quantile in June 2020.

Table 4 results yield that the governmental policies in response to the COVID-19 pandemic through governments’ restrictions on traditional (face-to-face) education, traditional daily social life, commerce, travel, and public and private sector services became successful to reduce the spread of COVID-19 disease.

The restrictions brought about distance education, social distancing, delays in travel, and closures of public and private working places, entertainment places, and wedding venues; church and mosque activities and other types of social gatherings were also restricted. For instance, according to Beck and Hensher (2020), the bans/restrictions due to the COVID-19 outbreak in Australia were successful through the government’s request to “stay at home.” The restriction policies, besides other privileges, targeted to impose some implementations on high-risk populations (e.g., older aged people and people having several infectious) to prevent the healthcare system from being overwhelmed by reducing especially the demand for intensive care units.

The article additionally analyzes the total deaths due to COVID-19 (Appendix Table A4). Appendix Table A4 has four emergent outcomes: (i) The number of COVID-19 cases is significant in determining the number of COVID-19 deaths; (ii) no matter what the age structure (the share of the median age, age 65, and age 70 in the population) is, it has positive significant impacts on COVID-19 deaths; (iii) the number of hospital beds has a negative significant effect on COVID-19 deaths; and (iv) GDP per capita has a negative significant effect on the number of deaths in the model in which median age is employed. In the models in which the age 65 and age 70 populations are employed, the income (per capita GDP) seems to be insignificant.

As GDP per capita increases, the COVID-19 death rate of the median age population gets lower. As the share of the median age population increases, the number of total deaths per million decreases. If hospital beds per thousand increased by 1 unit, total deaths per million will decrease by 1.84 units. Another reason for the increase in COVID-19 deaths is total cases per million. On the other hand, the findings show that GDP does not help people aged 65 and 70 and older to prevent death due to COVID-19. Considering especially the G20 countries, it might be observed that there is no common association between the values of per capita GDP and mortality. In some countries, per capita GDP and mortality rates from COVID-19 follow a positive association, while in some countries, these two variables move in the opposite direction.

For instance, taking into account the G20 countries with the highest income, while the mortality rates from COVID-19 are found relatively higher in the United States, the United Kingdom, and France, these rates are found relatively lower in Australia, Canada, Germany, and Japan (WHO, 2020b; World Bank, 2020).

On the other hand, the relationship can be evaluated in terms of diagnostic capabilities which refer to the equipment required at the diagnosis stage, such as equipment, testing, and laboratories (Aggarwal et al., 2015). For COVID-19, diagnostic tests (PCR and CRISPR) have stood out among these capabilities (Vandenberg et al., 2020). It might be stated that there is a heterogeneous structure between these tests representing diagnostic capabilities and GDP levels of countries. The total number of tests is subject to change from one high-income country to another high-income country. For instance, the tests realized in the United States, the United Kingdom, France, and Canada are 222 million, 47 million, 29 million, and 13 million, respectively. The number of tests implemented in countries with relatively low income might also vary significantly. For instance, the test numbers of China, India, Turkey, and Pakistan are 160 million, 155 million, 21 million, and 6 million, respectively (Statista, 2020).

## Discussion

Although different ages (median, 65, and 70+) have significant positive contributions to the number of total COVID-19 cases, the older population has a relatively more significant contribution to the number of cases due to some chronic illnesses that older people may have, such as heart disease, cancer, and diabetes.

The question is whether health administrators can follow more efficient policies that can lower the increasing rate of chronic illnesses. Health administrations, with greater allocated administrative budgets, can follow more efficient public health policies to reduce tobacco use and exposure to secondhand smoke and poor nutrition, including diets low in fruits and vegetables and high in sodium and saturated fats. Besides, relevant policies should be implemented to increase physical activity and to reduce excessive alcohol use in societies (CDC, 2020). It is also necessary to keep people away from the use of nonprescription drugs (Seyed-Hosseini et al., 2010).

The bans and/or restrictions due to the COVID-19 epidemic were found successful through the administrators’ policy to “stay at home.” However, one might ask if authorities considered also the adverse effects of the stringency policy. The government policies in response to the COVID-19 pandemic should consider also some potential adverse results such as depression, anxiety, loneliness, stigma, basic commodity insecurity, violence, and other adverse consequences in social life (Zhang et al., 2020b). There exist several articles in the COVID-19 literature regarding mental healthcare from a medical perspective (Teng et al., 2020). To reduce the adverse effect of restriction due to COVID-19, scientists, gerontologists, virologists, therapists, epidemiologists, and policymakers should consider simultaneously to reach a balance in public health (Golubev, 2020).

During the COVID-19 pandemic, the highest priority should be given to functional medical care and vital societal services. To prevent the public from additional physical and mental distress, government authorities, public health management services, and the public itself should consider maintaining physical activity (Jakobsson et al., 2020).

During the COVID-19 pandemic, the countries’ healthcare infrastructure becomes more important to reduce the fatalities caused by the outbreak. In this stage, societies and their representative authorities ought to give more priority to health and environmental issues than other domestic and foreign affairs/politics. Domestic and international actions should keep the current dialog on the role of domestic and global markets in sustainable development considering the quality environment and health (Crawford, 2006; Mihalache-O’Keef, 2018).

Continental differences in the number of high-risk groups, particularly the presence of older people in Europe and the Americas, may raise the question of whether these governments’ public health policies are ineffective. The recent experience postulates the existence of ineffective measures for suppressing and controlling transmission in Europe and the United States. These measures must be supported by financial and social programs that encourage community responses and address the inequities that have been amplified by the pandemic (Alwan et al., 2020). Thus, permanent restrictions will be required in the short-term to lessen transmission and fix ineffective pandemic response systems in order to prevent future lockdowns. By contrast, some countries like Japan, Vietnam, and New Zealand have shown that robust public health responses contribute positively to reduce transmission, allowing life to return to nearnormal. Besides, it is necessary to adopt measures that must change rapidly and fundamentally if these tensions are to be successfully managed, aimed to correct collective action, information asymmetries, irrationality, negative externalities, and the related free-riding phenomenon persistently distorting the Member States’ combined efforts, resulting in deficient attempts to contain the spread of COVID-19 (Kovac et al., 2020). Hence, the lack of institutional mechanisms, long-term planning, and willingness to invest in healthcare have all come to light with the COVID-19 outbreak (Peeri et al., 2020). In consequence, both regions, Europe and the United States, need to assume a well-coordinated policy response to limit economic, social, and health damage and re-establish financial stability (Regling, 2020).

## Limitations

We conducted multicollinearity tests for estimated models through VIF analyses. The findings indicate that all estimated models, except the model in which the independent variables are median age, age 65, and age 70 populations, do not suffer from multicollinearity. Multicollinearity needs to be considered seriously. On the other hand, the multicollinearity problem may not be a serious problem when *R*^2^ is high and individual *t* statistics are significant (Johnston, 1984). Blanchard (1987) also states that multicollinearity is essentially a data deficiency problem and sometimes researchers might have no choice over the available data for parameter estimations (Kennedy, 1998). However, considering multicollinearity a problem in estimations, further study might launch ridge regression and/or a combination of cross-sectional data and time series (pooled data) or drop some variables from the model to eliminate the multicollinearity issue.

Although our research conducted efficient panel model estimations, future researches might follow different methodologies such as panel spatial models to discover the direct and indirect effects of explanatory variables on COVID-19 cases. Our research employed 209 cross-sectional observations of April, June, and August 2020. Due to the lack of data for the stringency index in August, this article could not analyze the impact of government policies in response to the COVID-19 outbreak during August. Future works might explore the stringency index of August 2020 and analyze its effect on the spread of the COVID-19 pandemic. Future works can also consider analyzing the adverse effects of government policies in response to the COVID-19 epidemic (depression, anxiety, loneliness, and stigma) quantitatively.

## Conclusion

This article considers evaluating the potential determinants of the spread of COVID-19 pandemic disease in 209 countries around the world. This work has observed (a) the impacts of the shares of median age and older age population (age 65 and age 70 or older) on COVID-19 total cases, (b) the effect of stringency policy on COVID-19 cases, and (c) the influences of total COVID-19 cases, age structure, number of hospital beds, and income level on the number of total deaths due to COVID-19. The highlights and results of this research are as follows:

(i) GLM, GMM, and quantile regressions indicate that age structure is vital in determining the total number of cases in April, June, and August of 2020. The impact of age structure on COVID-19 shows a prominent increase from median age to age 70 and older people. According to quantile (75) regression analyses in August 2020, the effect of a unit increase in the age 65 population on total cases is three times greater than the effect of a unit increase in the median age population on total cases. The impact of the share of age 70 in the population on total cases is 1.6 times greater than that of the share of the age 65 population.
(ii) Stringency policies cover government policies inresponse to the COVID-19 outbreak such as school closures, workplace closures, travel bans, and others, which are found significant to lower COVID-19 cases during April and June 2020. The bans/restriction regulations should impose some additional practices on high-risk people (older people with chronic disease) to reduce the demand for intensive care units of healthcare systems.
(iii) The estimations also yield that the availability of more hospital beds can reduce the number of deaths stemming from the COVID-19 epidemic. This output indicates the necessity of more employment of physical/technical equipment and associated medical personnel, especially in intensive care units.
(iv) Except GLM estimations, GMM and quantile regression estimations assert that GDP per capita does not matter in both less developed and developed countries, which have been affected by the corona pandemic. This outcome might exhibit evidence that low-income countries as well as high-income countries couldnot prevent their societies from deaths due to COVID-19 disease.
(v) Continental-based findings indicate that higher-risk groups (with the presence of older people) in Europe and America are higher than those in Asia and Africa.

The outputs of this work might suggest that policymakers of both developed and less developed countries follow strict health policies to diminish the adverse effects of COVID-19 disease. The relevant health policies might be (a) the policy of staying at home; (b) if necessary, considering meetings only in hygienic places by observing social distancing efficiently and by wearing masks; (c) the policy of recommending/supporting the essential medicine and health products/technologies; (d) following healthy diabetes/eating/drinking modules/programs; (e) considering regular sport and exercises; (f) following distance education programs; (g) subsidizing e-commerce and/or shopping through media; and (g) implementing the policy for people aged 65 or over to follow strictly items (a), (b), (c), (d), (e), and (f). These suggestions have the potential to lower the level of undesired adverse effects of COVID-19 disease in the world.

Overall, this research paper underlines the fact that age 65 or 70 and older populations might affect prominently the undesired results of the pandemic. This article, hence, suggests that administrators follow the outcomes of this research to diminish the potential current and future influences of COVID-19 on the welfare of the societies through some efficient health, education, commerce, and social programs.

## Data Availability Statement

The original contributions presented in the study are included in the article/Supplementary Material, further inquiries can be directed to the corresponding author/s.

## Author Contributions

FB: conceptualization, methodology, data curation and analysis, visualization, project administration—supervision and coordination, writing, original draft preparation, reviewing, editing, and validation. MD: project administration—supervision and coordination, writing, original draft preparation, reviewing, editing, and validation. SK: software, conceptualization, analysis, project administration—coordination, writing, original draft preparation, reviewing, and editing. DL: investigation, conceptualization, analysis, project administration—coordination, writing, original draft preparation, reviewing, and editing. FÜ: data curation, original draft preparation, reviewing, editing, and validation. PG: writing, original draft preparation, and reviewing. EM: original draft preparation, reviewing, and editing. All authors contributed to the article and approved the submitted version.

## Conflict of Interest

The authors declare that the research was conducted in the absence of any commercial or financial relationships that could be construed as a potential conflict of interest.

## Appendix

**TABLE A1:** The variables and sources.

**Variable**	**Definition**	**Source**
Total_cases_per_ million	Total confirmed cases of COVID-19 per 1,000,000 people	European Centre for Disease Prevention and Control
Total_ deaths_per_million	Total deaths attributed to COVID-19 per 1,000,000 people	European Centre for Disease Prevention and Control
Population	Population in 2020	United Nations, Department of Economic and Social Affairs, Population Division, World Population Prospects: The 2019 Revision
Median_ age	The median age of the population, United Nation projection for 2020	United Nation Population Division, World Population Prospects, 2017 Revision
Aged-65_ older	Share of the population that is 65 years and older, most recent year available	World Bank – World Development Indicators, based on age/sex distributions of the United Nations Population Division’s World Population Prospects: 2017 Revision
Aged-70_ older	Share of the population that is 70 years and older in 2015	United Nations, Department of Economic and Social Affairs, Population Division (2017), World Population Prospects: The 2017 Revision
GDP_per_ capita	Gross domestic product at purchasing power parity (constant 2011 international dollars), most recent year available	World Bank – World Development Indicators, source from the World Bank, International Comparison Program database
Stringency_ index	Government Response Stringency Index: composite measure based on nine response indicators including school closures, workplace closures, and travel bans, rescaled to a value from 0 to 100 (100 = strictest response)	Oxford COVID-19 Government Response Tracker, Blavatnik School of Government
Hospital_beds_ per_ thousand	Hospital beds per 1,000 people, a most recent year available since 2010	OECD, Eurostat, World Bank, National Government records, and other sources

*OWID COVID-19-data, October 2020.*

**TABLE A2:** The list of the cross sections.

**Continent**	**Location**
Africa	Algeria, Angola, Benin, Botswana, Burkina Faso, Burundi, Cameroon, Cape Verde, Central African Republic, Chad, Comoros, Congo, Cote d’Ivoire, Democratic Republic of the Congo, Djibouti, Egypt, Equatorial Guinea, Eritrea, Ethiopia, Gabon, Gambia, Ghana, Guinea, Guinea-Bissau, Kenya, Lesotho, Liberia, Libya, Madagascar, Malawi, Mali, Mauritania, Mauritius, Morocco, Mozambique, Namibia, Niger, Nigeria, Rwanda, Sao Tome and Principe, Senegal, Seychelles, Sierra Leone, Somalia, South Africa, South Sudan, Sudan, Swaziland, Tanzania, Togo, Tunisia, Uganda, Western Sahara, Zambia, and Zimbabwe.
Asia	Afghanistan, Armenia, Azerbaijan, Bahrain, Bangladesh, Bhutan, Brunei, Cambodia, China, Georgia, India, Indonesia, Iran, Iraq, Israel, Japan, Jordan, Kazakhstan, Kuwait, Kyrgyzstan, Laos, Lebanon, Malaysia, Maldives, Mongolia, Myanmar, Nepal, Oman, Pakistan, Palestine, Philippines, Qatar, Saudi Arabia, Singapore, South Korea, Sri Lanka, Syria, Taiwan, Tajikistan, Thailand, Timor, Turkey, United Arab Emirates, Uzbekistan, Vietnam, and Yemen.
Europe	Albania, Andorra, Austria, Belarus, Lithuania, Luxembourg, Macedonia, Malta, Moldova, Monaco, Montenegro, Netherlands, Norway, Poland, Portugal, Romania, Russia, San Marino, Serbia, Slovakia, Slovenia, Spain, Sweden, Switzerland, Ukraine, United Kingdom, and Vatican.
North America	Anguilla, Antigua and Barbuda, Aruba, Bahamas, Barbados, Belize, Bermuda, Bonaire Sint Eustatius, and Saba, British Virgin Islands, Canada, Cayman Islands, Costa Rica, Cuba, Curacao, Dominica, Dominican Republic, El Salvador, Greenland, Grenada, Guatemala, Haiti, Honduras, Jamaica, Mexico, Montserrat, Nicaragua, Panama, Puerto Rico, Saint Kitts and Nevis, Saint Lucia, Saint Vincent and the Grenadines, Sint Maarten (Dutch), Trinidad and Tobago, Turks and Caicos Islands, United States, and United States Virgin Islands.
Oceania	Australia, Fiji, French Polynesia, Guam, New Caledonia, New Zealand, Northern Mariana Islands, and Papua New Guinea.
S. America	Argentina, Bolivia, Brazil, Chile, Colombia, Ecuador, Falkland Islands, Guyana, Paraguay, Peru, Suriname, Uruguay, and Venezuela.
	

**TABLE A3:** The impact of age structure on spread of COVID-19 total cases in continents generalized linear model (Newton-Raphson/Marquardt steps).

**Independent Variables**	**Coefficient**	**Standard error**	***z*-Statistic**	**Probability**
(In Europe)				
Median age	16.25	3.38	4.80	0.0000
Aged-65_older	38.01	7.96	4.77	0.0000
Aged-70_older	58.89	12.07	4.87	0.0000
(In Asia)				
Median age	2.86	0.68	4.19	0.0000
Aged-65_older	9.92	2.92	3.39	0.0007
Aged-70_older	14.18	4.44	3.18	0.0014
(In America)				
Median age	3.30	0.67	4.86	0.0000
Aged-65_older	11.68	2.00	5.81	0.0000
Aged-70_older	18.30	3.24	5.64	0.0000
(In Africa)				
Median age	0.64	0.13	4.78	0.0000
Aged-65_older	4.24	0.61	6.89	0.0000
Aged-70_older	7.02	1.04	6.74	0.0000

**TABLE A4:** The total deaths due to COVID-19 pandemic generalized linear model (Newton-Raphson/Marquardt steps).

**Independent variables**	**Coefficient**	**Standard error**	***z*-Statistic**	**Probability**
**Median age**	1.00	0.37	2.70	0.0068
Hospital beds per thousand	–1.84	0.98	–1.87	0.0608
GDP per capita	–2.74	1.09	–2.50	0.0124
Total cases per million	0.028	0.00	8.40	0.0000
**Aged-65_older**	1.14	0.41	2.73	0.0063
Hospital beds per thousand	–1.84	0.98	–1.88	0.0595
GDP per capita	–0.44	0.37	–1.19	0.2336
Total cases per million	0.02	0.00	7.67	0.0000
Aged-70_older	1.90	0.61	3.10	0.0019
Hospital beds per thousand	–2.08	0.98	–2.11	0.0342
GDP per capita	–0.41	0.35	–1.16	0.2432
Total cases per million	0.02	0.00	7.31	0.0000
