# Supplementary material for: The Age Structure, Stringency Policy, Income, and Spread of Coronavirus Disease 2019: Evidence From 209 Countries
Source: Front Psychol. 2021 Feb 12;11:632192. doi: 10.3389/fpsyg.2020.632192 (PMC7907165; doi:10.3389/fpsyg.2020.632192)
Supplement: Supplementary file 1 [file Data_Sheet_1.docx]

| **Table A1.** The Variables and Sources | | |
| --- | --- | --- |
| **Variable** | **Definition** | **Source** |
| Total_Cases_Per  Million | Total confirmed cases of COVID -19 per 1,000,000 people | European Centre for Disease Prevention and Control |
| Total_Deaths_Per  Million | Total deaths attributed to COVID -19 per 1,000,000 people | European Centre for Disease Prevention and Control |
| Population | Population in 2020 | United Nations, Department of Economic and Social Affairs, Population Division, World Population Prospects: The 2019 Revision |
| Median_Age | The median age of the population, UN projection for 2020 | UN Population Division, World Population  Prospects, 2017 Revision |
| Aged-65_Older | Share of the population that is 65 years and older, most recent year available | World Bank – World Development Indicators, based on age/sex distributions of the United Nations Population Division's World Population  Prospects: 2017 Revision |
| Aged-70_Older | Share of the population that is 70 years and older in 2015 | United Nations, Department of Economic and Social Affairs, Population Division (2017), World Population Prospects: The 2017 Revision |
| GDP_Per_Capita | Gross domestic product at purchasing power parity (constant 2011 international dollars), most recent year available | World Bank – World Development Indicators, source from the World Bank, International Comparison Program database |
| Stringency_Index | Government Response Stringency Index: composite measure based on 9 response indicators including school closures, workplace closures, and travel bans, rescaled to a value from 0 to 100 (100 = strictest response) | Oxford COVID-19 Government Response Tracker, Blavatnik School of Government |
| Hospital_Beds_Per  Thousand | Hospital beds per 1,000 people, a most recent year  available since 2010 | OECD, Eurostat, World Bank, national  Government records and other sources |

Owid [COVID-19-data](https://github.com/owid/covid-19-data), October 2020

**Table A2.** The list of the Cross Sections

| **Continent** | **Location** |
| --- | --- |
| Africa | Algeria, Angola, Benin, Botswana, Burkina Faso, Burundi, Cameroon, Cape Verde, Central African Rep., Chad, Comoros, Congo, Cote d'Ivoire, Dem. Rep. of Congo, Djibouti, Egypt, Equatorial Guinea, Eritrea, Ethiopia, Gabon, Gambia, Ghana, Guinea, Guinea-Bissau, Kenya, Lesotho, Liberia, Libya, Madagascar, Malawi, Mali, Mauritania, Mauritius, Morocco, Mozambique, Namibia, Niger, Nigeria, Rwanda, Sao Tome and Principe, Senegal, Seychelles, Sierra Leone, Somalia, South Africa, South Sudan, Sudan, Swaziland, Tanzania, Togo, Tunisia, Uganda, Western Sahara, Zambia, Zimbabwe. |
| Asia | Afghanistan, Armenia, Azerbaijan, Bahrain, Bangladesh, Bhutan, Brunei, Cambodia, China, Georgia, India, Indonesia, Iran, Iraq, Israel, Japan, Jordan, Kazakhstan, Kuwait, Kyrgyzstan, Laos, Lebanon, Malaysia, Maldives, Mongolia, Myanmar, Nepal, Oman, Pakistan, Palestine, Philippines, Qatar, Saudi Arabia, Singapore, South Korea, Sri Lanka, Syria, Taiwan, Tajikistan, Thailand, Timor, Turkey, U. Arab Emirates, Uzbekistan, Vietnam, Yemen |
| Europe | Albania, Andorra, Austria, Belarus, Lithuania, Luxembourg, Macedonia, Malta, Moldova, Monaco, Montenegro, Netherlands, Norway, Poland, Portugal, Romania, Russia, San Marino, Serbia, Slovakia, Slovenia, Spain, Sweden, Switzerland, Ukraine, United Kingdom, Vatican. |
| N. America | Anguilla, Antigua and Barbuda, Aruba, Bahamas, Barbados, Belize, Bermuda, Bonaire Sint Eustatius, and Saba, British Virgin Islands, Canada, Cayman Islands, Costa Rica, Cuba, Curacao, Dominica, Dominican Republic, El Salvador, Greenland, Grenada, Guatemala, Haiti, Honduras, Jamaica, Mexico, Montserrat, Nicaragua, Panama, Puerto Rico, Saint Kitts and Nevis, Saint Lucia, Saint Vincent and the Grenadines, S. Maarten (Dutch), Trinidad and Tobago, Turks and Caicos Islands, United States, US Virgin Islands. |
| Oceania | Australia, Fiji, French Polynesia, Guam, New Caledonia, New Zealand, N. Mariana Islands, Papua New Guinea. |
| S. America | Argentina, Bolivia, Brazil, Chile, Colombia, Ecuador, Falkland Islands, Guyana, Paraguay, Peru, Suriname, Uruguay, Venezuela. |

**Table A3.** The Impact of Age Structure on Spread of COVID-19 Total Cases in Continents

Generalized Linear Model (Newton-Raphson / Marquardt steps)

| **Independent Var.** | Coefficient | Std. Error | z-Statistic | Prob. |
| --- | --- | --- | --- | --- |
| **(In Europe)** |  |  |  |  |
| *Median Age* | 16.25 | 3.38 | 4.80 | 0.0000 |
| *Aged-65_Older* | 38.01 | 7.96 | 4.77 | 0.0000 |
| *Aged-70_Older* | 58.89 | 12.07 | 4.87 | 0.0000 |
| **(In Asia)** |  |  |  |  |
| *Median Age* | 2.86 | 0.68 | 4.19 | 0.0000 |
| *Aged-65_Older* | 9.92 | 2.92 | 3.39 | 0.0007 |
| *Aged-70_Older* | 14.18 | 4.44 | 3.18 | 0.0014 |
| **(In America)** |  |  |  |  |
| *Median Age* | 3.30 | 0.67 | 4.86 | 0.0000 |
| *Aged-65_Older* | 11.68 | 2.00 | 5.81 | 0.0000 |
| *Aged-70_Older* | 18.30 | 3.24 | 5.64 | 0.0000 |
| **(In Africa)** |  |  |  |  |
| *Median Age* | 0.64 | 0.13 | 4.78 | 0.0000 |
| *Aged-65_Older* | 4.24 | 0.61 | 6.89 | 0.0000 |
| *Aged-70_Older* | 7.02 | 1.04 | 6.74 | 0.0000 |

**Table A4.** The Total Deaths due to COVID-19 Pandemic

**Generalized Linear Model (Newton-Raphson / Marquardt steps)**

| **Independent Variables** | **Coefficient** | **Std. Error** | **z-Statistic** | **Prob.** |
| --- | --- | --- | --- | --- |
| ***Median Age*** | 1.00 | 0.37 | 2.70 | 0.0068 |
| *Hospital Beds Per Thousand* | -1.84 | 0.98 | -1.87 | 0.0608 |
| *GDP Per Capita* | -2.74 | 1.09 | -2.50 | 0.0124 |
| *Total Cases Per Million* | 0.028 | 0.00 | 8.40 | 0.0000 |
| ***Aged-65_Older*** | 1.14 | 0.41 | 2.73 | 0.0063 |
| *Hospital Beds Per Thousand* | -1.84 | 0.98 | -1.88 | 0.0595 |
| *GDP Per Capita* | -0.44 | 0.37 | -1.19 | 0.2336 |
| *Total Cases Per Million* | 0.02 | 0.00 | 7.67 | 0.0000 |
| *Aged-70_Older* | 1.90 | 0.61 | 3.10 | 0.0019 |
| *Hospital Beds Per Thousand* | -2.08 | 0.98 | -2.11 | 0.0342 |
| *GDP Per Capita* | -0.41 | 0.35 | -1.16 | 0.2432 |
| *Total Cases Per Million* | 0.02 | 0.00 | 7.31 | 0.0000 |
